# Changes in glycemic control before and after COVID-19 quarantine

**DOI:** 10.17843/rpmesp.2023.403.12830

**Published:** 2023-09-25

**Authors:** Javier R. Murillo-Valle, Juan L. Meza-Ponte

**Affiliations:** 1 Vitarte Hospital, Lima, Peru. Vitarte Hospital Lima Peru

To the Editor. In Peru, the first imported case of COVID-19 was confirmed on March 5, 2020. On March 15, a state of national emergency was declared and mandatory social isolation was implemented. Different studies have shown that countries that have quarantined their populations caused interruption of care and delay of treatment ^1^. In our country, the quarantine modified habits, increasing sedentarism and carbohydrate consumption. These changes may have altered glycemic control (GC) in patients with type 2 diabetes *mellitus* (DM2).

The UKPDS study demonstrated the benefits of adequate GC in reducing the prevalence of microvascular complications ^2^; likewise, all guidelines recommend an individual and multifactorial approach to the different cardiovascular risk factors; for example, healthy lifestyle changes, emotional balance and appropriate medication under medical supervision are the basis for adequate control ^3^^,^^4^. These were altered in quarantine.

Reports of GC during quarantine were mixed. It is suggested that the impact on GC will differ according to the country and type of diabetes. No studies related to GC were found in Peru. For this reason, we conducted an observational study to evaluate GC in patients with DM2 before and after quarantine due to COVID-19. This research was approved and authorized by the Teaching and Research Unit and by the Ethics Committee of the Hospital De Vitarte, with approval code: No. 03-2023-CIEI/HV.

According to the American Diabetes Association (ADA), good GC is defined as glycosylated hemoglobin (Hb1Ac) under 7% ^3^. Outpatient visits to the endocrinology service were defined as “C1” when they took place pre-quarantine between July 2019 to February 2020 and “C2” when they took place post-quarantine between July 2022 to September 2022. A total of 232 patients were selected in C2 using non-probabilistic sampling by convenience and data of the same patients during C1 were obtained from the medical records. We selected a sample of 88 patients who met the following inclusion criteria: being diagnosed with DM2 six months prior to quarantine and being older than 18 years of age. Those hospitalized in the last six months before the blood test took place, those with oncologic disease or another type of diabetes, those who had an analysis outside the institution or those with incomplete data were excluded.

We included variables such as age, sex, time of disease and diabetic parameters such as basal glucose, HbA1c, BMI, systolic blood pressure (SBP), diastolic blood pressure (DBP), total cholesterol, HDL, LDL, triglycerides, and creatinine. HbA1c was considered as the basis for the definition of GC ^5^. Differences in the means between C1 and C2 were evaluated generally.

Mean and standard deviation were used to describe the variables. We used Student’s t-test for related samples during analytical analysis. A value of p<0.05 was considered statistically significant. All calculations were performed with the STATA 17 software.

The characteristics of the C1 sample can be found in the supplementary material. Most were women (69.3%), the mean age was 60.86 (SD: 9.81) years. the mean time of illness was 12.43 (SD: 8.36) years, mean fasting glucose was 161 ± 66 mg/dl and mean HbA1c was 8.70% (SD: 2.41). A significant increase in mean HbA1c was found after the quarantine due to COVID-19 (C1: 8.70 vs. C2: 9.42, p=0.005). We also noticed a significant increase in serum LDL. Other covariates did not show significant differences (Table 1).

**Table 1:** Difference in glycemic control and covariates before (C1) and after (C2) the quarantine due to COVID-19.

Variables	C1 Mean ± SD	C2 Mean ± SD	p-value
HbA1C	8.70 ± 2.40	9.42 ± 2.70	0.005
Glucose (mg/dl)	161.09 ± 66.40	181.14 ± 82.20	0.066
BMI (kg/m^2^)	28.67 ± 5.40	29.44 ± 5.80	0.058
SBP (mmHg)	125.30 ± 15.80	128.00 ± 17.00	0.273
DBP (mmHg)	74.30 ± 9.50	73.90 ± 10.80	0.799
Cholesterol (mg/dl)	187.32 ± 58.50	192.70 ± 47.00	0.357
LDL (mg/dl)	96.49 ± 38.70	107.83 ± 34.20	0.012
HDL (mg/dl)	48.31 ± 10.40	50.65 ± 9.90	0.115
Triglycerides (mg/dl)	170.38 ± 131.80	186.35 ± 91.90	0.071
Creatinine (mg/dl)	0.73 ± 0.17	0.75 ± 0.25	0.496

C1: Outpatient visits to the endocrinology service from July 2019 to February 2020.

C2: Outpatient visits to the endocrinology service between July 2022 and September 2022.

HbA1c: glycosylated hemoglobin, BMI: body mass index, SBP: systolic blood pressure, DBP: diastolic blood pressure, SD: standard deviation,

Previous studies on the impact of quarantine on GC in DM2 have reported different results. Tannus *et al*. observed that glycosylated hemoglobin values decreased after insulin use ^6^. Psoma *et al*. found a significant decrease in glycemia during confinement ^7^. An increase in HbA1c was reported in India after three weeks of quarantine, with similar findings in China and Korea ^8^. This may be explained by factors such as psychological stress and difficulty in obtaining medication and accessing medical care. A systematic review and meta-analysis found an increase in HbA1c levels ^9^. These results are similar to those obtained by our study.

One of the limitations of this study is that the sample was not very large; however, it is similar to previous studies that compared the glycemic control of patients with DM2 before and after quarantine by COVID-19. Data collection, in retrospect, decreased our sample. In addition, we did not adjust the analysis for variables such as therapy, comorbidities, or lifestyle changes, which may be confounding factors because they can influence GC.

In conclusion, a significant increase in HbA1c and LDL was found in patients with DM2 after quarantine by COVID-19. This study shows an increase in HbA1c possibly due to decreased access to health care. Further research is needed to strengthen the evidence reported by this study.
