# Supplementary material for: Changes in glycemic control before and after COVID-19 quarantine
Source: Rev Peru Med Exp Salud Publica. 2023 Sep 25;40(3):372–3. doi: 10.17843/rpmesp.2023.403.12830 (PMC10953671; doi:10.17843/rpmesp.2023.403.12830)
Supplement: Supplementary material. — Available in the electronic version of the RPMESP. [file rpmesp-40-03-12830-s001.docx]

**MATERIAL SUPLEMENTARIO**

Anexo 1. Características antes de la cuarentena (C1) de los pacientes con DM2.

| **Variables** | **Mínimo** | **Máximo** | **Media** | **DE** |
| --- | --- | --- | --- | --- |
| Edad (Años) | 34 | 84 | 60.86 | 9.81 |
| Tiempo enfermedad | 2 | 40 | 12.43 | 8.36 |
| G. ayunas (mg/dl) | 76.1 | 334 | 161.09 | 66.04 |
| HbA1c (%) | 5.3 | 15 | 8.70 | 2.41 |
| Peso (kg) | 44 | 110 | 68.22 | 13.72 |
| IMC (kg/m^2^) | 17.78 | 45.78 | 28.67 | 5.41 |
| PAS (mmHg) | 98 | 178 | 125.31 | 15.83 |
| PAD (mmHg) | 56 | 106 | 74.34 | 9.58 |
| Colesterol (mg/dl) | 76 | 362 | 187.32 | 58.50 |
| HDL (mg/dl) | 18.4 | 95.6 | 48.31 | 10.44 |
| LDL (mg/dl) | 32 | 194.8 | 96.49 | 38.86 |
| Triglicéridos (mg/dl) | 40.3 | 958 | 170.38 | 121.86 |
| Creatinina (mg/dl) | 0.42 | 1.18 | 0.74 | 0.17 |

DE: Desviación estándar
