# Supplementary material for: Changes in glycemic control before and after COVID-19 quarantine
Source: Rev Peru Med Exp Salud Publica. 2023 Sep 25;40(3):372–3. doi: 10.17843/rpmesp.2023.403.12830 (PMC10953671; doi:10.17843/rpmesp.2023.403.12830)
Supplement: Translation

# Translation: Cambios en el control glucémico antes y después de la cuarentena por la COVID-19

**Authors:** Javier R. Murillo-Valle, Juan L. Meza-Ponte

**Affiliations:** 1 Vitarte Hospital, Lima, Peru.

Sr. Editor. En el Perú, el 5 de marzo del 2020 se confirmó el primer caso importado de COVID- 19. El 15 de marzo es declarado el estado de emergencia nacional disponiéndose el aislamiento social obligatorio. Diferentes estudios demostraron que los países que dispusieron la cuarentena en sus poblaciones produjeron la interrupción de las atenciones y el retraso de los tratamientos ^1^. En nuestro país, la cuarentena modificó las costumbres generando un aumento en el sedentarismo y el consumo de carbohidratos. Estos cambios pudieron haber alterado el control glucémico (CG) en los pacientes con diabetes *mellitus* tipo 2 (DM2).

El estudio UKPDS demostró los beneficios del adecuado CG al reducir la prevalencia de las complicaciones microvasculares ^2^; así mismo, todas las guías recomiendan un enfoque individual y multifactorial sobre los diferentes factores de riesgo cardiovascular; por ejemplo, los cambios a un estilo de vida saludable, un equilibrio emocional y la medicación adecuada bajo la supervisión médica son la base de un adecuado control ^3^^,^^4^. Estos fueron alterados en la cuarentena.

Los reportes del CG durante la cuarentena fueron variados. Se plantea que el impacto en el CG será diferente según el país y el tipo de diabetes. No se encontraron estudios relacionados al CG en el Perú. Por tal razón, se realizó un estudio observacional con el objetivo evaluar el CG en pacientes con DM2 antes y después de la cuarentena por la COVID-19. El estudio fue aprobado y autorizado por la Unidad de Docencia e Investigación y por el Comité de ética del Hospital De Vitarte, con código de aprobación: N.° 03-2023-CIEI/HV.

Según la American Diabetes Association (ADA), el buen CG se define como una hemoglobina glicosilada (Hb1Ac) con valores por debajo del 7% ^3^. Las visitas al consultorio externo de Endocrinología se definieron como “C1” cuando fue realizada previa a la cuarentena entre julio del 2019 a febrero del 2020 y como “C2” a la realizada luego de la cuarentena entre julio del 2022 a setiembre del 2022. Se seleccionaron a 232 pacientes en C2 mediante un diseño no probabilístico por conveniencia, los datos de C1 de los mismos pacientes se obtuvieron de las historias clínicas. Se seleccionó una muestra de 88 pacientes que cumplieron los siguientes criterios de inclusión: tener diagnóstico de DM2 seis meses antes de la cuarentena y ser mayores de 18 años. Se excluyeron a los hospitalizados en los últimos seis meses a la toma de análisis, a quienes presentaron enfermedad oncológica u otro tipo de diabetes, que tenían un análisis fuera de la institución o aquellos con datos incompletos.

Se incluyeron variables como la edad, sexo, tiempo de enfermedad y parámetros diabéticos como la glucosa basal, HbA1c, IMC, presión arterial sistólica (PAS), presión arterial diastólica (PAD), colesterol total, HDL, LDL, triglicéridos, creatinina. Se considero a la HbA1c como base para la definición de CG ^5^. Se evaluaron las diferencias de las medias entre C1 y C2 de forma general.

Se utilizo la estadística descriptiva para el cálculo de medias y desviación estándar. Con respecto a la estadística analítica; se utilizó la prueba de T de Student para muestras relacionadas. Se consideró un valor de p<0,05 como estadísticamente significativo. Todos los cálculos se realizaron con el programa estadístico Stata 17.

Las características de la muestra en C1 se muestran en el material suplementario. La mayoría fueron mujeres (69,3%); la media de edad fue de 60,86 (DE: 9,81) años; el tiempo de enfermedad fue de 12,43 (DE: 8,36) años, la glucosa en ayunas fue de 161 ± 66 mg/dl y la HbA1c fue de 8,70% (DE: 2,41). Se encontró un incremento significativo en la media de la HbA1c antes y después de la cuarentena por la COVID-19 (C1: 8,70 *vs.* C2: 9,42, p=0,005). También se encontró el incremento significativo de LDL sérico. En los demás parámetros diabéticos no se encontraron diferencias significativas (Tabla 1).

**Tabla 1:** Diferencia en el control glucémico y covariables antes (C1) y después (C2) de la cuarentena por la COVID-19.

Variables	C1 Media ± DE	C2 Media ± DE	Valor de p
HbA1C	8,70 ± 2,40	9,42 ± 2,70	0,005
Glucosa (mg/dl)	161,09 ± 66,40	181,14 ± 82,20	0,066
IMC (kg/m^2^)	28,67 ± 5,40	29,44 ± 5,80	0,058
PAS (mmHg)	125,30 ± 15,80	128,00 ± 17,00	0,273
PAD (mmHg)	74,30 ± 9,50	73,90 ± 10,80	0,799
Colesterol (mg/dl)	187,32 ± 58,50	192,70 ± 47,00	0,357
LDL (mg/dl)	96,49 ± 38,70	107,83 ± 34,20	0,012
HDL (mg/dl)	48,31 ± 10,40	50,65 ± 9,90	0,115
Triglicéridos (mg/dl)	170,38 ± 131,80	186,35 ± 91,90	0,071
Creatinina (mg/dl)	0,73 ± 0,17	0,75 ± 0,25	0,496

C1: visita al consultorio externo de Endocrinología entre julio del 2019 a febrero del 2020

C2: visita al consultorio externo de Endocrinología entre julio del 2022 a setiembre del 2022.

HbA1c: Hemoglobina glicosilada, IMC: Índice de masa corporal, PAS: Presión arterial sistólica, PAD: Presión arterial diastólica, DE: Desviación estándar.

Diferentes estudios que evaluaron el impacto de la cuarentena en el CG en la DM2 reportaron resultados variables. Tannus *et al*. observaron que los valores de hemoglobina glicosilada disminuyeron luego del uso de insulina ^6^. Psoma *et al*. descubrieron una disminución significativa en la glicemia, durante el confinamiento ^7^. En India se informó un incremento de los valores de HbA1c después de tres semanas de cuarentena, con hallazgos similares en China y Corea ^8^. Esto puede ser explicado por factores como el estrés psicológico y a la dificultad para obtener medicamentos y atención médica. Una revisión sistemática y metaanálisis se encontró un incremento en los niveles de HbA1c ^9^. Estos resultados son similares a los obtenidos por nuestro estudio.

Como limitación del estudio se debe reconocer que la muestra no fue muy grande; sin embargo, es similar a estudios previos que compararon el control glicémico de pacientes con DM2 antes y después de la cuarentena por la COVID-19. La obtención de datos, en retrospectiva, disminuyó nuestra muestra. No se realizó un análisis ajustado por variables como la terapia, comorbilidades o cambios en el estilo de vida, las cuales pueden ser factores de confusión debido a que pueden influir en el CG.

En conclusión, se encontró un incremento significativo de la HbA1c y LDL en pacientes con DM2 después de la cuarentena por la COVID-19. Este estudio muestra un incremento de los valores de HbA1c posiblemente por un menor acceso a la atención de salud. Se necesitan más investigaciones para fortalecer la evidencia generada por este estudio.
